# Rescue of mutant rhodopsin traffic by metformin-induced AMPK activation accelerates photoreceptor degeneration

**DOI:** 10.1093/hmg/ddw387

**Published:** 2016-11-30

**Authors:** Dimitra Athanasiou, Monica Aguila, Chikwado A. Opefi, Kieron South, James Bellingham, Dalila Bevilacqua, Peter M. Munro, Naheed Kanuga, Francesca E. Mackenzie, Adam M. Dubis, Anastasios Georgiadis, Anna B. Graca, Rachael A. Pearson, Robin R. Ali, Sanae Sakami, Krzysztof Palczewski, Michael Y. Sherman, Philip J. Reeves, Michael E. Cheetham

**Affiliations:** 1 UCL Institute of Ophthalmology, 11-43 Bath Street, London, UK; 2 School of Biological Sciences, University of Essex, Wivenhoe Park, Essex, UK; 3 Moorfields Eye Hospital NHS Trust, 162 City Road, London, UK; 4 Department of Pharmacology, and Cleveland Center for Membrane and Structural Biology, School of Medicine, Case Western Reserve University, 10900 Euclid Avenue, Cleveland, OH, USA; 5 Department of Biochemistry, Boston University Medical School, Boston, Massachusetts, MA, USA

## Abstract

Protein misfolding caused by inherited mutations leads to loss of protein function and potentially toxic ‘gain of function’, such as the dominant P23H rhodopsin mutation that causes retinitis pigmentosa (RP). Here, we tested whether the AMPK activator metformin could affect the P23H rhodopsin synthesis and folding. In cell models, metformin treatment improved P23H rhodopsin folding and traffic. In animal models of P23H RP, metformin treatment successfully enhanced P23H traffic to the rod outer segment, but this led to reduced photoreceptor function and increased photoreceptor cell death. The metformin-rescued P23H rhodopsin was still intrinsically unstable and led to increased structural instability of the rod outer segments. These data suggest that improving the traffic of misfolding rhodopsin mutants is unlikely to be a practical therapy, because of their intrinsic instability and long half-life in the outer segment, but also highlights the potential of altering translation through AMPK to improve protein function in other protein misfolding diseases.

## Introduction

Inherited mutations in rhodopsin (*RHO*), the light sensitive protein of rod photoreceptor cells, are the most common cause of autosomal dominant retinitis pigmentosa (adRP). Rhodopsin is the archetypal G protein-coupled receptor (GPCR) comprising a 348 amino acid apoprotein rod opsin covalently linked with the vitamin A derived chromophore 11-*cis*-retinal (1). Rod opsin protein is an integral membrane protein with an N-terminus that is located intradiscally (corresponding to the extracellular location of other GPCRs), a C-terminus that is an intracellular and seven transmembrane (TM) helices (2). Most rhodopsin mutations, such as P23H the most common mutation in North America, are found in the intradiscal or transmembrane domains of rhodopsin and cause rhodopsin misfolding (Class II mutations) (3). An understanding of the molecular mechanisms underlying Class II rhodopsin mutants came from cell culture models which showed that misfolded rod opsin is retained in the endoplasmic reticulum (ER), as opposed to wild-type (WT) normal protein which translocates to the plasma membrane. These results suggested the existence of an ER quality control machinery that does not permit further traffic of misfolded rod opsin (4,5). Misfolded rod opsin is retrotranslocated from the ER and degraded via the ubiquitin proteasome system; however, non-degraded mutant rod opsin can spontaneously aggregate and form intracellular inclusions (6,7). Mutant rod opsin can also exert a dominant-negative effect on the processing of WT rod opsin (6,8) and can activate the unfolded protein response (UPR) (9).

In photoreceptors, correctly folded rod opsin is transported from the inner segments (IS) to the rod outer segments (ROS) and inserted in densely packed disks. Proper folding of rod opsin is a prerequisite for visual transduction since the co-ordination of the natural ligand 11*-cis*-retinal within the fully folded opsin generates the rhodopsin photopigment. Apart from abolishing rhodopsin function, and of greater pathological significance, misfolding also leads to photoreceptor cell death. In animal models, P23H rhodopsin is localised primarily to rod IS and outer nuclear layer (ONL) and ER stress and dominant-negative mechanisms have been observed (9–11). However, some P23H can traffic to the ROS whereby retinal degeneration is accelerated by light (12,13). Another potential disease mechanism is the destabilisation of ROS architecture (10,14,15). For example, in the P23H knock-in (KI) mouse model, over 90% of mutant rhodopsin is degraded, whereas the remaining protein can escape the ER and traffic to the ROS where it disrupts ROS disk formation, affecting stability and resulting in photoreceptor vulnerability and death (14–16).

The high metabolic demand and complex architecture of the retina requires strict regulation of proteostasis (protein homeostasis) for protection against several insults, including those arising from protein misfolding (reviewed in (17)). Similar to other neurodegenerative diseases, the toxic-gain-of-function effect of mutant rhodopsin, could also reflect the incapacity of photoreceptors to maintain proteostasis. Therefore, a number of therapeutic approaches have attempted to manipulate proteostasis mechanisms to reduce rhodopsin aggregation, enhance the degradation of mutant rhodopsin, stimulate autophagy or correct misfolding. For instance, pharmacological chaperones, kosmotropes (or chemical chaperones) and molecular chaperone inducers have been found to counteract some of the effects of the P23H mutation in cells (6,18–21).

Additionally, drugs such as arimoclomol and Hsp90 inhibitors that stimulate the cell stress responses in the retina, can protect against photoreceptor cell death in transgenic rats expressing P23H rhodopsin by reducing P23H aggregation (22,23). In contrast, pharmacological chaperones, such as 11- and 9-*cis*-retinal, can improve P23H rod opsin folding in cells if they are present during rod opsin biogenesis (6,19,20). Though vitamin A supplementation improved the survival of rod cells in T17M rhodopsin transgenic mice (24), 11-*cis*-retinal is highly abundant in the retina and this approach has not been reported in P23H animal models.

An alternative way to enhance folding and reduce aggregation of mutant proteins could be to target polypeptide chains early in their synthesis on the ribosome. Studies have shown that codon usage bias correlates with protein secondary structure through an effect on the translation elongation rate (25). Different tRNAs have different concentrations in the cell resulting in slower ribosome recognition for the rare tRNAs and causing translational pausing. By slowing down peptide formation in critical regions, the folding machinery is able to process the protein more efficiently to improve overall folding. For instance, an improvement was observed in mutant cystic fibrosis transmembrane conductance regulator (CFTR) trafficking following a reduction in the elongation speed due to a triplet deletion (26). Furthermore, in another study, mild inhibition of protein translation via pharmacological treatments improved the folding of mutant CFTR and other polypeptides (27–29).

Protein translation is tightly controlled by adenosine 5’-monophosphate activated protein kinase (AMPK), an enzyme which acts as a sensor of cellular energy status by monitoring the ratio of AMP:ATP and is referred to as the master regulator of energy homeostasis (30). Two main mechanisms have been proposed through which AMPK can regulate protein translation: activation of the eukaryotic elongation factor 2 kinase (eE2K) which phosphorylates its target eukaryotic elongation factor 2 (eEF2) leading to inhibition of the elongation step; and inhibition of the mammalian target of rapamycin (mTOR), a serine/threonine kinase that regulates the initiation step. The combination of these mechanisms leads to changes in the initiation and elongation phases of mRNA translation (30,31).

Several drugs, including metformin, which is the first-line treatment for type II diabetes and the most widely prescribed antidiabetic drug, can activate AMPK. Metformin suppresses excessive hepatic glucose production, improves insulin sensitivity and also protects against the cardiovascular complications of diabetes as it inhibits lipid biosynthesis (32). Metformin reportedly also has an anti-tumour effect and has been suggested as a potential treatment for cancer and lymphoma (33–35).

In this study, we used a pharmacological approach to investigate the potential effect of mild inhibition of protein translation on mutant rod opsin disease mechanisms. Metformin treatment improved P23H rod opsin folding and trafficking and reduced P23H-related cell death in cell culture models; however, the improvement in P23H traffic after metformin treatment *in vivo* resulted in the reduced photoreceptor cell function and increased photoreceptor cell death. Taken together, these results suggest that metformin can improve rhodopsin folding and trafficking, but this structural rescue is transient and eventually enhances the instability of rod outer segments.

## Results

### Metformin enhances P23H rod opsin trafficking

The folding of mutant CFTR can be improved by inhibitors of protein translation, elongation or initiation (28). The long-term use of such translational inhibitors, however, is limited because prolonged inhibition of protein translation is toxic (27). Therefore, we tested the ability of the AMPK activators, metformin and 5-amino-1-β-D-ribofuranosyl-imidazole-4-carboxamide (AICAR), to partially reduce translation and investigate the hypothesis that mild inhibition of protein translation can improve the traffic of misfolded rod opsin.

**Figure 1:**
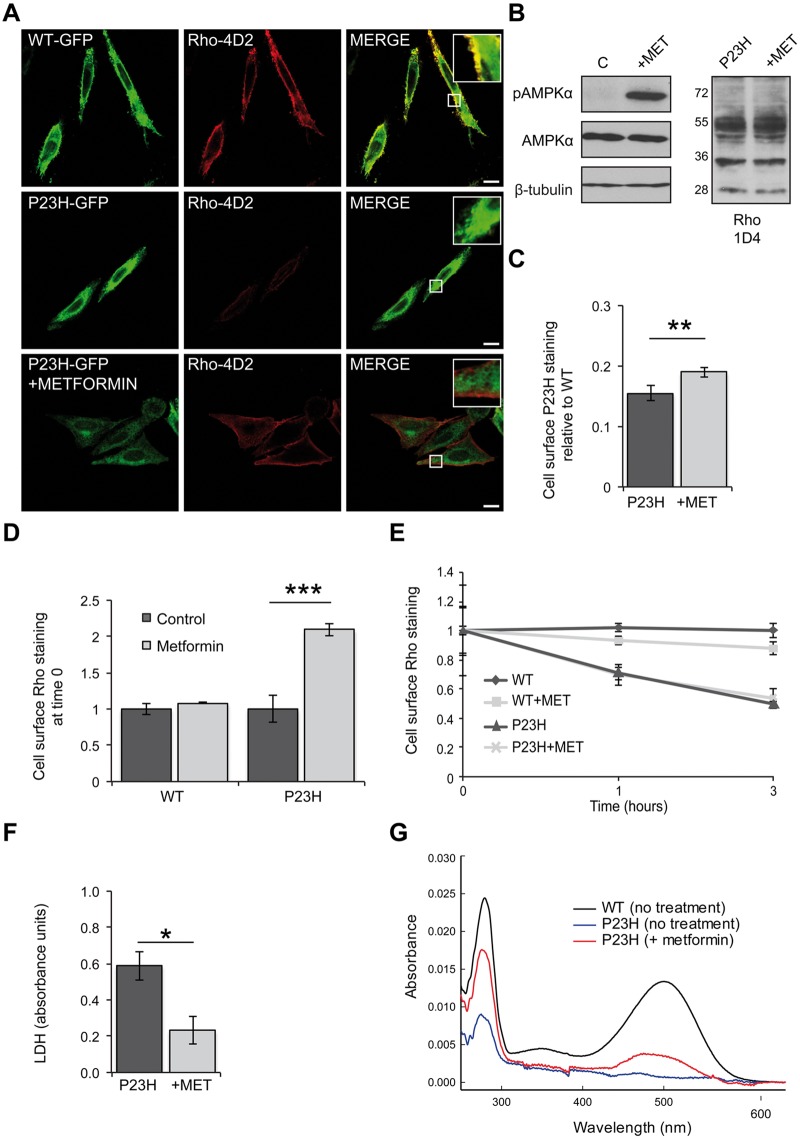
Metformin improves P23H rod opsin traffic and folding and reduces P23H-induced cell death. (**A**) SK-N-SH cells transfected with WT-GFP rod opsin (green) or P23H-GFP rod opsin (green) were treated with metformin (1 mM) for 18 h. Fixed, non-permeabilised cells were stained with Rho-4D2 antibody against the extracellular N-terminus (red). Confocal microscopy imaging under identical conditions. Scale bar: 10 μm. Boxed regions show higher magnification. (**B**) Immunoblot with an anti-phospho-AMPKα (p-AMPKα) or an anti-AMPKα antibody. β-Tubulin was used as a loading control. Untreated- (C) and metformin- (+MET) treated P23H-GFP cells were blotted with the Rho-1D4 antibody. (**C**) In-cell western analysis of HA-P23H rod opsin. SK-N-SH cells were fixed and immunostained with an HA antibody against the extracellular N-terminus of rod opsin. The non-permeabilised (cell surface) immunoreactivity was determined as a percentage of total permeabilised immunoreactivity. The data were normalised to the amount of cell surface HA-WT rod opsin, values ± SEM, *n ≥* 4, **P < 0.01, unpaired two-sided Student's *t* test. (**D-E**) Rhodopsin internalisation assay in HA-WT or HA-P23H SK-N-SH untreated cells or metformin treated (18 h). Live cells were incubated with an HA antibody for 30 min at 37 °C, washed, media returned and rod opsin was allowed to internalise for 0, 1 and 3 h. (**D**) Cell surface rod opsin staining at time 0. Values ± SEM, *n ≥* 3, ***P < 0.001, unpaired two-sided Student's *t* test. (**E**) Remaining cell surface WT or P23H rod opsin staining after 0, 1 and 3 h of internalisation in the presence or absence of metformin, normalised to time 0. Values ± 2SEM, *n ≥* 3. (**F**) LDH assay on cells expressing P23H-GFP rod opsin treated with 1 mM metformin (+MET). Average absorbance values ± SEM, *n ≥* 3, *P < 0.05, unpaired two-sided Student's *t* test. The LDH positive control (100% cell death) was 2.3 absorbance units. **(G)** UV-visible absorption spectra of immunoaffinity purified WT (black) and P23H (blue) rhodopsin pigments obtained from tetracycline inducible HEK293S cell lines treated with 300 μM metformin (red) for 24 h during expression The WT sample was diluted to show the profile on a similar scale.

The effect of metformin was initially investigated on SK-N-SH cells expressing P23H-GFP rod opsin. Following 18 h of treatment, non-permeabilised cells were stained with the Rho-4D2 antibody, which has an epitope in the extracellular domain, to reveal the P23H-GFP that had trafficked to the cell surface (Fig. 1A). Metformin treatment resulted in a general reduction of P23H-GFP rod opsin intracellular levels compared to untreated cells and an increase in Rho-4D2 staining on the plasma membrane. The same effect was also observed after treatment with AICAR, and both compounds enhanced AMPK phosphorylation (Fig. 1B and Supplementary Material, Fig. S1). The enhanced traffic of P23H rod opsin in the presence of metformin and AICAR was confirmed by in-cell western analyses of permeabilised and non permeabilised HA-P23H transfected cells stained with the HA antibody which targets the extracellular domain of rod opsin. (Fig. 1C and Supplementary Material, Fig. S1). Both metformin and AICAR also improved P23H rod opsin traffic in HEK293S cells (Supplementary Material, Fig. S2).

To assess the turn-over of rod opsin at the plasma membrane and whether this could be affected by metformin to alter cell surface staining, we used a pulse of HA antibody labelling on non-permeabilised, live cells and monitored the cell surface expression of WT and P23H rod opsin at different time points of chase (Fig. 1D and E). At time 0, WT rod opsin cell surface staining remained unchanged after metformin treatment while P23H cell surface staining was significantly increased (Fig. 1D). P23H rod opsin was more rapidly removed from the membrane, almost 50% after 3 h, than WT rod opsin, which was relatively stable (Fig. 1E). In both cases, metformin did not significantly affect this turnover suggesting that the increased cell surface expression of P23H rod opsin at the membrane is due to increased traffic and not altered turnover (Fig. 1E).

Expression of P23H rod opsin in SK-N-SH cells results in enhanced cell death (19). Using a lactate dehydrogenase (LDH) assay, we confirmed this finding with P23H-GFP expressing cells. Importantly, after 48 h treatment with metformin (Fig. 1F) or AICAR (Supplementary Material, Fig. S1) a significant reduction in the level of cytotoxicity compared to the untreated cells was observed. These data suggest AMPK activation could also protect these cultured cells from the toxic effects associated with the P23H mutation.

The effect of metformin and AICAR on the activity of mTOR, a downstream effector of AMPK that modulates protein translation, was investigated. Immunoblot analysis of the phosphorylation status of downstream targets of mTOR involved in translation, such as S6 Ribosomal Protein, phospho-p70 S6 Kinase, phospho-eIF4B and 4E-BP1, showed reduction of phosphorylation upon metformin and AICAR treatment (Supplementary Material, Fig. S1). This would correlate with inhibition of mTOR signalling and a potential reduction in translation, as previously described for AMPK activation (36,37).

Emetine has been shown to improve CFTR folding through mild inhibition of protein synthesis (28); therefore, emetine was investigated to test if it also promotes P23H traffic to the plasma membrane. In-cell western analysis showed that emetine could also significantly increase P23H traffic to the plasma membrane (Supplementary Material, Fig. S1). These data suggest that inhibition of translation elongation increases P23H rod opsin traffic; however, emetine was not as effective as metformin, thus we concentrated the rest of the study on metformin.

The improved traffic of P23H-GFP after metformin treatment suggested enhanced folding of the protein in the ER, allowing it to escape ER quality control. To assess this possibility, we tested the ability of P23H rod opsin to form functional pigment to directly assess folding. UV-visible-absorption spectroscopy was performed for P23H rod opsin purified from a tetracycline inducible HEK293S stable cell line (38) in the presence or absence of metformin during biogenesis. Metformin treatment resulted in 6.4 fold increase in P23H pigment formation (Fig. 1G), indicating that in this model mild inhibition of translation improves both the folding and trafficking of mutant rhodopsin.

Metformin appeared to have no effect on the traffic of WT rhodopsin. Moreover, no difference was observed in WT rhodopsin thermal stability, photobleaching profile or metarhodopsin decay when metformin was present during WT rhodopsin expression, or when added to purified rhodopsin, confirming that WT rhodopsin is not affected by metformin treatment (Supplementary Material, Fig. S3). These data also suggest that the effect of metformin on mutant rhodopsin traffic was not caused by direct binding to rhodopsin, but is mediated by altering folding during biogenesis.

### Metformin treatment enhances P23H trafficking in the P23H knock-in mouse model

**Figure 2:**
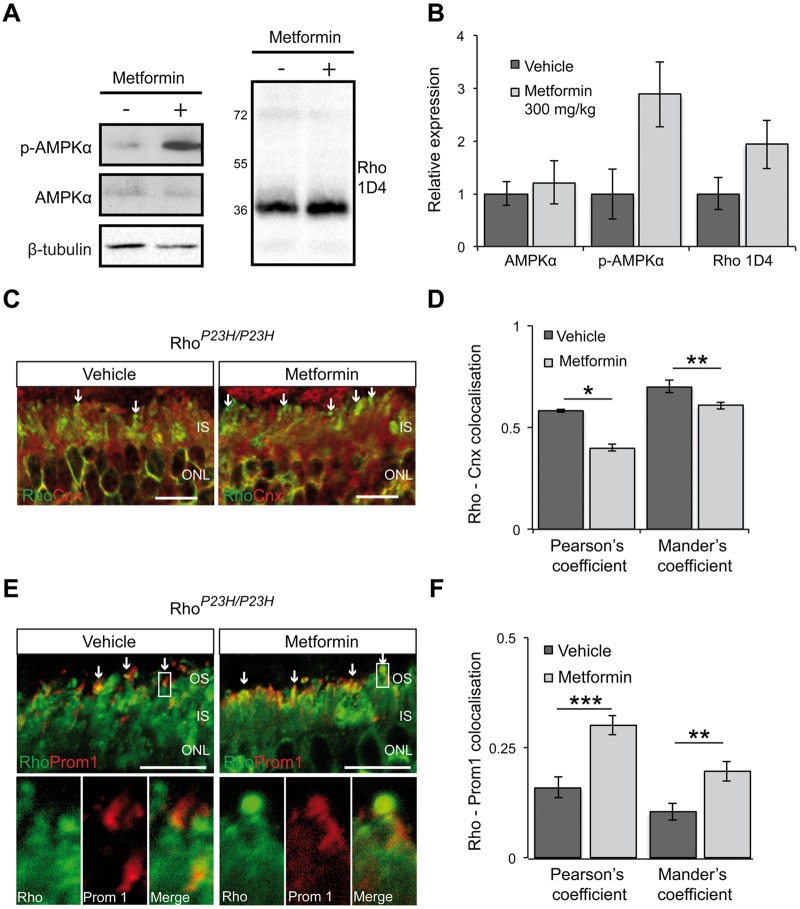
Increased levels of P23H in the ROS of Rho^P23H/P23H^ mice after metformin treatment. (**A**) Retinal extracts from metformin- (+) or vehicle- treated (-) Rho*^P23H/P23H^*(P14) KI mice were western blotted with anti-rhodopsin Rho-1D4 antibody and p-AMPKα or anti-AMPKα antibody. β-Tubulin was used as a loading control. (**B**) Quantification of p-AMPKα and rhodopsin levels in the Rho^*P23H/P23H*^ KI mouse retina relative to β-tubulin. Densitometric analysis was used to calculate the levels of rhodopsin in metformin-treated mice relative to vehicle; values are means ± SEM, *n*≥3 biological replicates. (**C**) Subcellular localisation of rhodopsin (green) and the ER marker calnexin (red) in the retina from Rho^*P23H/P23H*^ KI mice treated with either vehicle-PBS or metformin. Scale bar 10 µm. **(D**) Rhodopsin-calnexin IS staining quantified by calculating the Pearson's and Mander's co-localisation co-efficients using the JaCOP plug-in and ImageJ software, *n = *18 images each from 2 vehicle-treated mice and 2 metformin-treated mice. Values are means ± SEM, *P < 0.05, **P < 0.01, unpaired two-sided Student's *t* test. (**E**) Subcellular localisation of rhodopsin (green) in Rho^*P23H/P23H*^ KI mice retina treated with vehicle or metformin in relation to prominin 1 (red). Arrows highlight small OS, boxed area shown in close up below. Scale bar: 10 µm. **(F**) Rhodopsin-prominin 1 (Prom1) staining was quantified by calculating the Pearson's and Mander's co-localisation co-efficients using the JaCOP plug-in and ImageJ software, *n = *9 technical replicates/condition from 2 biological/condition. Values are means ± SEM, **P < 0.01, ***P < 0.001, unpaired two-sided Student's *t* test.

To confirm that metformin was able to affect P23H synthesis and traffic in photoreceptors, we used the homozygous P23H KI (Rho^*P23H/P23H*^) mouse that does not express WT rhodopsin. Since antibody probes cannot differentiate between P23H and WT rhodopsin, this would ensure any observed effect was specific to P23H rhodopsin. These mice undergo rapid retinal degeneration; prior to which they express low levels of rhodopsin and do not elaborate normal ROS disks (14,15). Therefore, an increase in rhodopsin expression and/or ROS assembly would reveal the effect of metformin specifically on P23H synthesis, degradation and trafficking. Daily treatment of Rho^*P23H/P23H*^ mice from P9 to P14 with metformin led to activation of AMPK in the mouse retina, which was accompanied by an increase in the rhodopsin protein level (Fig. 2A and B). Moreover, examination of the subcellular localisation of P23H rhodopsin showed that P23H was localised in needle-like structures at the top of the IS, as previously described (15), and in the IS of both untreated and treated mice (Fig. 2C). Treatment with metformin appeared to result in less IS staining and an increased localisation at the needle-like structures, which were increased in size. Indeed, co-localisation studies using Pearson’s and Mander’s coefficients and the ER marker calnexin showed significant reduction in co-localisation of the two proteins in the IS of metformin-treated retina compared to vehicle-treated retina (Fig. 2C and D), whereas staining with prominin-1 that is localised at the base of ROS, showed a significant increase of co-localisation between P23H rhodopsin and prominin-1 in metformin-treated retina (Fig. 2E and F). Collectively, these data show that metformin stimulates P23H rhodopsin trafficking in the Rho^*P23H/P23H*^ mouse model.

### Metformin impairs photoreceptor function and survival in P23H rodent models

**Figure 3:**
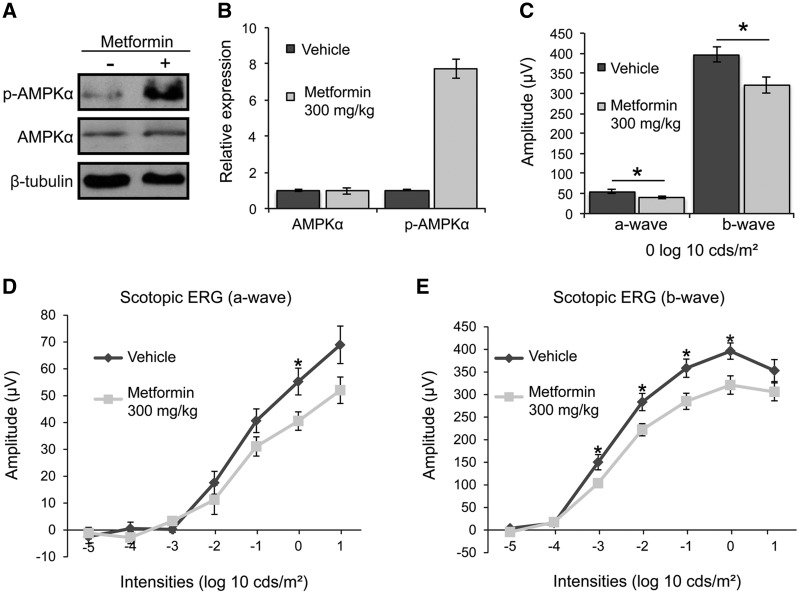
Metformin impairs photoreceptor function in P23H-1 rats. P23H-1 rats were treated from P21-P35 with either 300 mg/kg metformin or vehicle-PBS administered daily via IP injection. **(A**) Retinae of metformin- (+) or vehicle- treated (-) P23H-1 rats were western blotted with p-AMPKα or an anti-AMPKα antibody to confirm the activation of AMPKα protein in the rat retina after metformin treatment, as indicated. β-Tubulin was used as a loading control. (**B**) Quantified expression levels of AMPKα and p-AMPKα in P23H-1 retina relative to β-tubulin. Densitometric analysis was used to calculate the levels of AMPKα and p-AMPKα relative to the vehicle control; values are means ± SEM, *n* ≥ 3 biological replicates. **(C-E**) Scotopic ERG responses; (**C**) average at 0 log cds/m^2^, (**D**) a-wave, (**e**) b-wave of P23H-1 rats (P36) treated with either 300 mg/kg metformin (*n = *16 biological replicates) or vehicle-PBS (*n = *14 biological replicates), values are means ± SEM. *P < 0.05, unpaired two-sided Student's *t* test.

As these data showed that metformin could improve mutant P23H rod opsin traffic both *in vitro* and *in vivo* and improve cell viability in cell culture, we next investigated the effect of metformin in the P23H-1 transgenic rat. This model undergoes a rapid retinal degeneration with a 25% photoreceptor loss by postnatal day 15 (P15) as compared to WT rats (39). Despite this rapid decline in number of photoreceptors, we had previously identified a therapeutic window between P21 to P35 to test the ability of drugs to improve retinal function and preserve photoreceptor survival (22,23). Therefore, rats were treated daily from P21 to P35 with 300 mg/kg metformin by intraperitoneal (IP) injection. Previously, it was shown that a relatively high dose of metformin must be administered for rodents to achieve plasma metformin concentrations similar to those found in humans and to produce a therapeutic effect in a diabetic state (40–42). At P36, following two weeks of treatment, rats were anaesthetised with an IP injection of ketamine/xylazine before they were subjected to full field scotopic electroretinography (ERG) for assessment of visual function. Metformin treatment produced increased AMPK phosphorylation in rat retina (Fig. 3A and B). Surprisingly, photoreceptor function was decreased by metformin treatment, as evidenced by lower ERG responses with both scotopic a-wave and b-wave response amplitudes being reduced compared to those of vehicle-treated animals (Fig. 3C–E).

**Figure 4:**
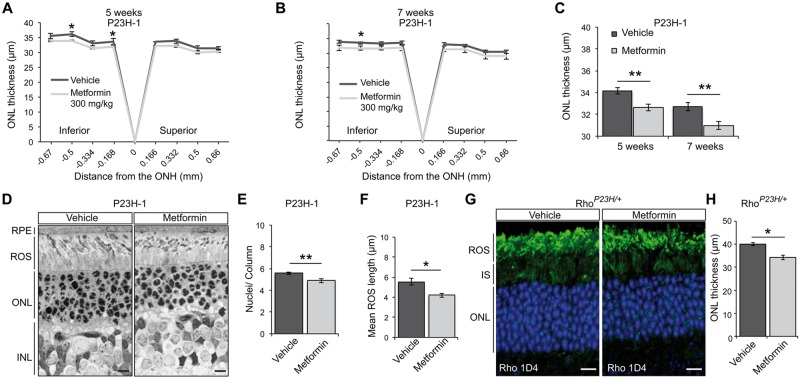
Metformin treatment accelerates photoreceptor loss in P23H rodent models. (**A-C**) ONL thickness of P23H-1 rats treated from P21-P35 (**A**) or from P21-P49 (**B**) with either 300 mg/kg metformin or vehicle-PBS daily via IP injection. P23H-1 ONL thickness at P36 (**A**) for metformin-treated (*n = *8 biological replicates) or vehicle-PBS treated rats (*n = *6 biological replicates) or P49 (**B**) (7 weeks of age) for metformin-treated (*n = *5 biological replicates) or vehicle-PBS treated rats (*n = *5 biological replicates) as assessed by OCT measurements across the inferior-superior meridian. Results are either expressed as a spider plot from the optic nerve head (ONH) (**A-B**) or as mean ONL thickness across the whole retina (**C**). Values are means ± SEM. **P < 0.01, unpaired two-sided Student's *t* test. (**D**) Representative images of semi-thin resin retinal sections from P23H-1 (P36) rats treated with vehicle or metformin. Sections were stained with toluidine blue. Scale bar: 10 μm. (**E-F)** Quantification of (**E**) number of nuclei/column across the retina of vehicle- or metformin-treated P23H-1 rats at P36 (*n* ≥ 4 biological replicates). (**F**) ROS length analysis was assessed within five images of ≥ 20 ROS across the retina from ≥3 animals per treatment. Values are means ± SEM. *P < 0.05; **P < 0.01, unpaired two-sided Student's *t* test. (**G**) Representative retinal images of P23H heterozygous KI (*Rho^P23H/+^*; P45) mice treated with vehicle or metformin, as indicated. Subcellular localisation of rhodopsin stained with Rho 1D4 antibody. Nuclei stained with DAPI. Scale bar: 10 µm. (**H**) Quantification of mean *Rho^P23H/+^* ONL thickness across the whole retina of 4 animals per treatment. Values are means ± SEM. *P < 0.05.

Optical coherence tomography (OCT) was used to image the retinal layers of vehicle- and metformin-treated rats and measure the thickness of the outer nuclear layer (ONL) as a marker for photoreceptor survival. As reported previously, the superior retina of P23H-1 rats was more damaged than the inferior (43); however, metformin treatment caused a reduction in the thickness of the ONL in the inferior retina compared to the ONL thickness of vehicle-treated rats (Fig. 4A). A longer treatment with metformin up to 7 weeks of age (P21–P49) confirmed a reduction in ONL thickness (Fig. 4B) and a significant reduction in the average ONL thickness in metformin-treated rats (Fig. 4C). These results suggest that metformin accelerates photoreceptor loss in the P23H-1 rats at a relatively constant rate and indicates that the increase is approximately equivalent to two weeks of degeneration.

To confirm the OCT reductions, we examined the ONL in metformin- and vehicle-treated eyes at P36 in semi-thin resin-embedded sections (Fig. 4D). Quantitation of the number of photoreceptor nuclei per column of the ONL in the 6 mm long retina showed a significant reduction of photoreceptor numbers in metformin-treated rats (Fig. 4E). This was accompanied with a significant reduction of the ROS length (Fig. 4F). Overall, these data suggest that metformin treatment results in enhanced photoreceptor loss accompanied by a reduction in P23H-1 visual function.

This effect of metformin on P23H rhodopsin expressing photoreceptors was confirmed by treating heterozygous P23H KI mice (*Rho^P23H/+^*). These mice have the same gene dosage as affected human RP individuals and are potentially a more accurate model without any overexpression artefacts, but they have been less widely used than the P23H-1 rats. Histological analyses revealed that treatment with metformin from P21 to P45 led to a significant decrease in ONL thickness (Fig. 4G and H), confirming that metformin enhances retinal degeneration.

To exclude the possibility that the effect of metformin is a result of general ocular toxicity in the retina, Sprague Dawley (SD) WT rats were treated daily with 300 mg/kg metformin or vehicle from P21 to P35 daily and ERG and OCT tests were performed at P36. Similar to the treatment of P23H-1 rats, metformin treatment enhanced AMPK phosphorylation in the retina (Supplementary Material, Fig. S4A and B). However, this was not accompanied with a reduction in the ERG responses, as both a-wave and b-wave amplitude responses between metformin and vehicle-treated rats were indistinguishable (Supplementary Material, Fig. S4C and D). Additionally, metformin did not affect photoreceptor survival up to P36 since no difference in the ONL thickness was observed (Supplementary Material, Fig. S4E and F).

These data showed that metformin does not affect a healthy retina. To confirm the toxicity was specific to rhodopsin, and rule out the possibility that metformin could accelerate photoreceptor cell death in the presence of another cause of retinal degeneration, we investigated the effect of metformin in the rhodopsin knock out (KO) (*Rho^−/−^*) mouse model that does not elaborate ROS and its photoreceptors die within three months (44). Mice were treated daily with metformin or vehicle from P21 to P35 and their cone visual function and photoreceptor survival was addressed at P36 by performing photopic ERG and OCT, respectively (Supplementary Material, Fig. S4G–I). Metformin treatment did not affect photoreceptor function (Supplementary Material, Fig. S4G) or survival (Supplementary Material, Fig. S4H and I), confirming that the adverse effects of metformin in the P23H-1 rat model and P23H KI mouse model are specific to rhodopsin and not a general feature associated with retinal degeneration.

### Metformin reduces the unfolded protein response (UPR) in the retina of P23H-1 rats

**Figure 5:**
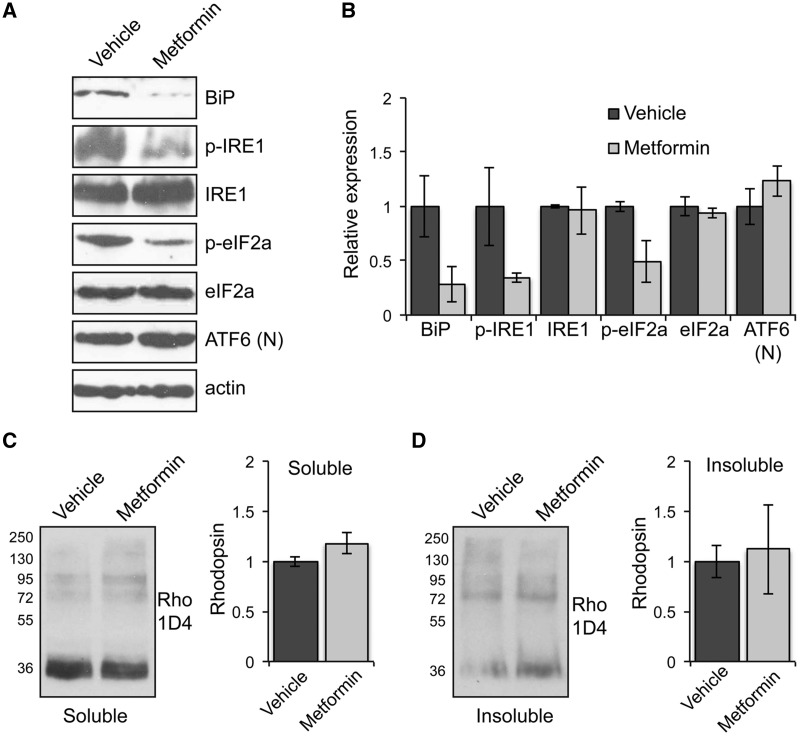
Metformin reduces the unfolded protein response (UPR) and does not affect the levels of rhodopsin in the retina of P23H-1 rats. **(A**) Retinae of P23H-1 rats treated from P21-P35 with either 300 mg/kg metformin or vehicle-PBS were analysed with markers of the three UPR branches and blotted with antibodies against BiP, p-IRE1, IRE1, p-eIF2A, eIF2a, and ATF6 cleaved or nuclear (N). Actin was used as a loading control. (**B**) Densitometric analysis was used to calculate the levels of BiP, p-IRE1, IRE1, p-eIF2A, eIF2a, and ATF6 (N) relative to vehicle after normalisation to actin; values are means ± SEM, *n* ≥ 3 (biological replicates). (**C** and **D**) Retinae of P23H-1 rats treated from P21-P35 with either 300 mg/kg metformin or vehicle-PBS were analysed by a sedimentation assay. Fractions were immunoblotted with the 1D4 antibody against rhodopsin. Densitometric analysis was used to calculate the levels of (C) soluble rhodopsin relative to the vehicle treated and (D) insoluble rhodopsin relative to the vehicle after normalisation to soluble rhodopsin. Values are means ± SEM, *n* ≥ 4 (biological replicates).

The ER retention of mutant rhodopsin induces the unfolded protein response (UPR) in P23H-1 rats (9,23). Therefore, we investigated whether metformin could ameliorate the ER stress associated with P23H misfolding. Western blot analysis showed that the induction of IRE1 and PERK branches of the UPR were reduced in metformin-treated rats, while there was no change in the induction of the ATF6 branch (Fig. 5A and B). These data in combination with an accompanied reduction in the BiP levels support a reduction in ER stress, which would correlate with an improvement in rhodopsin folding upon metformin treatment and potentially also alleviate any reduction in translation associated with PERK activation.

Furthermore, a sedimentation assay of the retinae of metformin and vehicle-treated P23H-1 rats showed no major differences in soluble (Fig. 5C) or insoluble (Fig. 5D) rhodopsin fractions, suggesting that metformin treatment did not have a significant effect on the rhodopsin expression level or aggregation.

### Improvement of P23H trafficking after metformin treatment is associated with disorganised rod outer segments (ROS)

**Figure 6:**
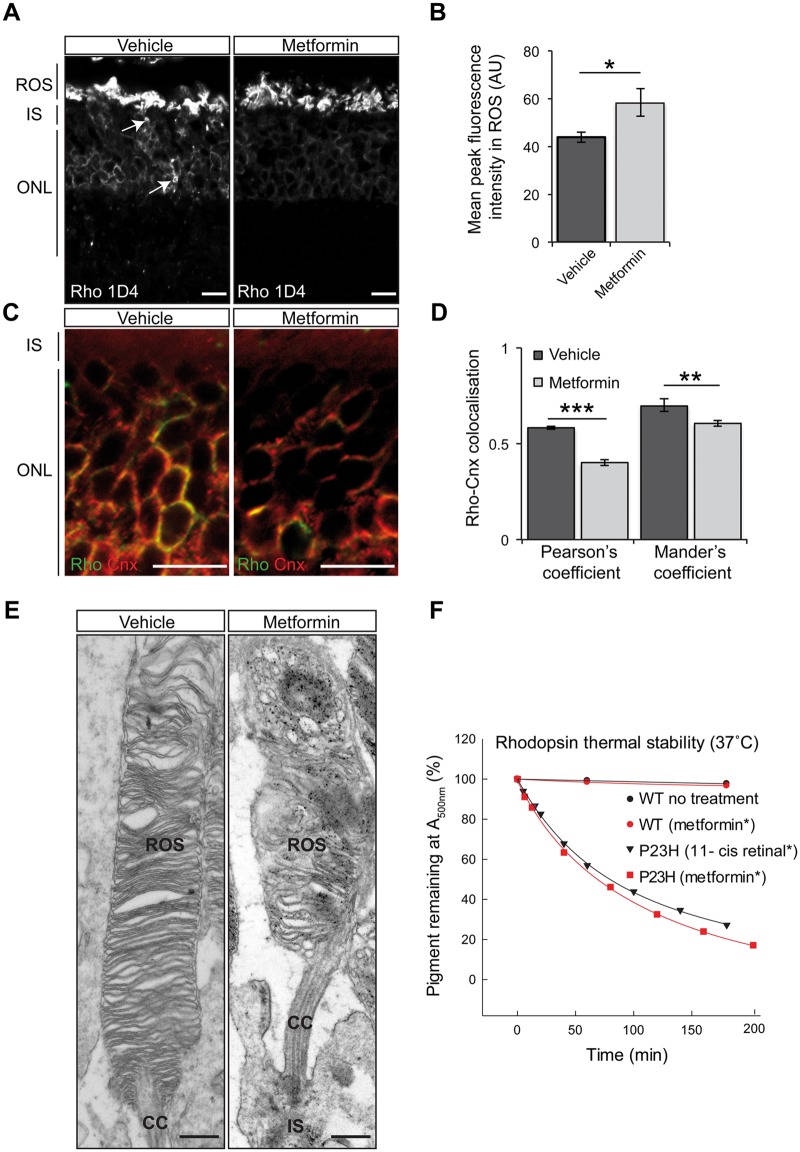
Improvement of rhodopsin trafficking correlates with disorganised ROS in the retinae of P23H-1 metformin-treated rats. (**A**) Representative retinal images of P23H-1 (P36) rats treated with vehicle or metformin, as indicated. Subcellular localisation of rhodopsin stained with Rho 1D4 antibody. Scale bar: 10 µm. (**B**) Quantification of rhodopsin immunofluorescence intensity in the ROS. A line scan was performed and the mean maximum intensity was assessed within 12 images of ≥ 15 ROS from three animals per treatment. Values are means ± SEM, *P < 0.05. (**C**) ONL staining of rhodopsin (green) and the ER marker, calnexin (red). Scale bar 10 µm. **(D**) Rhodopsin-calnexin ONL overlap was quantified by calculating the Pearson's and Mander's co-localisation co-efficients, using the JaCOP plug-in and ImageJ software, *n = *15 images each from two vehicle-treated rats and two metformin-treated rats. Values are means ± SEM, **P < 0.01, ***P < 0.001, unpaired two-sided Student's *t* test. (**E**) Representative TEM images of vehicle- and metformin-treated P23H-1 retina at P36 illustrating the structure of ROS. Scale bar: 500 nm. ROS, rod outer segment; CC, connecting cilium/cilia; IS, photoreceptor cell inner segment. (**F**) Rhodopsin thermal stability at 37 °C. Purified WT or P23H mutant pigments from transfected HEK-293S cells following biosynthetic treatment with either metformin (red circles or red squares) or 11-*cis* retinal treatment (black triangles). WT pigment from non-treated cells (black circles). Pigments were held at 37 °C (equilibrated for 2 min) before commencing repeated scans by UV-visible absorbance spectroscopy (650–250 nm) at 30 min intervals for at least 4 h. Spectra were normalised at 650 nm and 280 nm and the change in A500 nm was plotted as a function of time. All data were fitted with exponential decay function using SigmaPlot 12 in order to determine half-life values.

To further investigate rhodopsin localisation, retinae of vehicle- and metformin-treated P23H-1 rats were subjected to immunohistochemical analysis at P36. In metformin-treated rats, rhodopsin was mainly localised in ROS with less staining observed around the nuclei in cell bodies of the ONL compared to vehicle-treated rats (Fig. 6A). Furthermore, quantification of fluorescence intensity in the ROS revealed a significant increase in rhodopsin in the ROS, supporting improved traffic (Fig. 6B). The potential improvement of mutant rhodopsin trafficking or enhanced degradation of mutant ER-retained rhodopsin was further investigated by staining with the ER marker calnexin (Fig. 6C). In the untreated retina, calnexin co-localised with rhodopsin in the ER. This co-localisation was significantly reduced in metformin-treated rats as measured with Pearson’s and Mander’s coefficients suggesting that more rhodopsin trafficked beyond the ER after metformin treatment (Fig. 6D). The overall level of rhodopsin was not significantly altered (Fig. 5C and D), suggesting that a reduction in rhodopsin caused by the loss of photoreceptors (Fig. 4E) might be offset by an increase of rhodopsin in the remaining rods, and there is no enhanced degradation of rhodopsin.

Studies in the P23H KI mouse model have shown disk disorganisation due to the presence of P23H rhodopsin in ROS, suggesting that disk destabilisation could be a potential mechanism of photoreceptor degeneration (14,15). To explore the possibility that improved traffic of mutant rhodopsin might further compromise ROS structure, transmission electron microscopy (TEM) was used to analyse the ROS in more detail at P36. TEM revealed that metformin-treated P23H-1 rats had shorter, more fragmented ROS and more disoriented and loosely packed disks compared to their vehicle-treated controls (Fig. 6E).

P23H rod opsin is intrinsically unstable (45,46). Even though its folding can be partially rescued with 11- or 9-*cis* retinal added during biogenesis (6,19,20), this mutant pigment remains unstable and defective (46). Thus, we predicted that the metformin-rescued P23H rhodopsin would also be unstable. Our analyses of the thermal stability of P23H rod opsin synthesised in metformin treated cells revealed that the reconstituted protein pigment was as unstable as the 11-*cis-*retinal rescued P23H pigment (Fig. 6F, Table 1).

**Table 1:** Thermal stability of WT and P23H mutant pigments in the presence or absence of metformin during biogenesis.

Half life (t_1/2_) of thermal decay (min)		
**Mutant pigment**	**37 °C**	**56.5 °C**
WT no treatment	8920.1	37.6
WT 300 µM metformin	8192.9	38.7
P23H 10 µM 11-*cis* retinal	88.8	―
P23H 300 µM metformin	75.3	―

## Discussion

The manipulation of proteostasis represents a potential tool for the treatment of misfolded protein diseases, such as misfolded rod opsin based RP. The general aim is to improve protein folding, inhibit aggregation or promote degradation of proteins. Among these strategies, correcting protein folding has been the most challenging. Although pharmacological chaperones such as retinoids or non-isomerisable retinoid analogues can assist the trafficking and folding of P23H rod opsin in cell culture systems (18,19,47), they have limitations *in vivo*. One of the drawbacks is that 11-*cis* retinal is already present at high levels in the retina and increasing the amount of retinoid availability, in combination with light exposure could result in artificially high levels of all-*trans* retinal, which is toxic to photoreceptors (48). Retinoids also lose efficacy when bleached and since they do not discriminate between opsins any locked forms could potentially affect cone opsin function. A clinical trial of high dose vitamin A supplementation produced modest clinical improvements in RP patients (49). These studies were done in non-genotyped RP patients, so it is unclear whether these included patients with P23H rhodopsin, other rhodopsin mutations, or patients with other retinal dystrophies. Moreover, studies in the VPP mouse model of P23H RP showed that retinal degeneration was slower when variants of retinal isomerase RPE65 that produce lower levels of 11-*cis* retinal were present, suggesting that high levels of retinoids or retinal analogues might actually be deleterious for such forms of RP (50).

To investigate an alternative method of promoting P23H folding, we tested whether altering the rate of translation could reduce the production of misfolded polypeptides during biogenesis, by providing the P23H intradiscal domain with more time to fold properly and allow the mutant protein to exit from the ER (27,28). Indeed, overnight treatment of P23H rod opsin cells cultured with the AMPK activators, metformin or AICAR, or the translation elongation inhibitor emetine improved P23H trafficking to the plasma membrane. The AMPK activators reduced the phosphorylation of S6, S6K and eIF4B presumably through the mTOR signalling pathway, which could lead to altered rates of protein translation. Importantly, the improved traffic of mutant rod opsin correlated with an enhanced formation of folded photopigment. These results are in line with the study by Meriin and colleagues, who showed that mild inhibition of protein translation by emetine can improve the folding of mutant ΔF508-CFTR (28), but this is the first time is has been shown for AMPK activators. Previously, it was shown that autophagy inducers such as rapamycin, which also negatively regulates the mTOR pathway, could reduce P23H inclusion incidence and cell death, but failed to promote the translocation of P23H to the plasma membrane (19), suggesting that metformin and rapamycin act via different mechanisms, as reported in other studies (51).

Thus, we tested the potential of metformin to promote P23H folding in the P23H-1 rat model as a therapeutic strategy to slow down retinal degeneration. Despite some controversy about the ability of metformin to cross the blood brain barrier (52,53), systemic administration of metformin to rats and mice led to activation of AMPK in the retina suggesting that it can cross the blood retina barrier, as noted after subcutaneous injections of metformin in an animal model of endotoxin-induced uveitis (EIU) (54). But, in contrast, to the protective effect of metformin in the P23H cell culture model, *in vivo* treatment with metformin exacerbated photoreceptor cell death across the retina in P23H-1 rats and P23H KI mice. Additionally, impairment of visual function was greater in metformin-treated P23H-1 rats. Importantly, the same dose range and duration of treatment in SD rats and rhodopsin KO mice did not produce any defects in either visual function or photoreceptor survival, suggesting that metformin was acting exclusively through P23H rhodopsin. Since the cell culture model lacks outer segments, we investigated whether metformin mediates retinal degeneration due to a non-ER mechanism that affects ROS. Destabilisation of ROS has been proposed as a potential mechanism for photoreceptor degeneration caused by a small amount of defective rhodopsin that traffics to ROS in the P23H KI mouse model (14). Additionally, P23H rhodopsin has been reported to escape the ER and undergo time-dependent aggregation in the ROS of *Xenopus*, which in turn destabilises rod disks possibly due to the inherent instability of the mutant rhodopsin (55). In both cases it is likely that pharmacological chaperone rescue by the 11-*cis*-retinal present in the retina contributes to pigment formation and traffic by the mutant rod opsin (56). Similarly, we observed shorter and more disorganised and disorientated ROS in metformin-treated rats. Moreover, photoreceptor survival was particularly reduced in the inferior hemisphere of the retina that receives more light. This suggests that the fraction of P23H that escapes the ER quality control and traffics to ROS after treatment is still defective, unstable and cytotoxic especially when this P23H pigment is photobleached.

Upon purification, the metformin rescued P23H was intrinsically unstable, just like 11-*cis* retinal-rescued mutant rhodopsin and it is likely that once successfully transported to ROS its stability becomes further compromised by light exposure. Thus, enhanced folding of P23H leading to its exit from the ER and incorporation in the disks could exacerbate the toxic effects observed at the level of the ROS, as seen in other animal models (14,15,55).

Despite the pharmacological rescue of some adRP mutants with retinoids, studies have shown that purified mutant pigments are intrinsically unstable, exhibiting abnormal photobleaching, metarhodopsin decay and signalling properties (21,46). Metformin treatment did not affect the folding, photobleaching profile or metarhodopsin decay of WT rhodopsin, suggesting no direct effect or binding of metformin to rhodopsin. Therefore, the partial rescue of P23H folding is more likely due to modulation of protein translation, or other pleiotropic effects, and not due to pharmacological chaperone rescue. Several studies reported neuroprotection following pharmacologically mediated AMPK activation in several neurodegenerative conditions such as Parkinson’s disease, Huntington’s disease and multiple sclerosis (57–60). Furthermore, studies on diabetic retinopathy and EIU showed that activation of AMPK reduced retinal inflammation (54,61,62), and that AMPK in *Drosophila* protects photoreceptors from excitotoxicity and is required to maintain neuronal integrity (63). Therefore, the protective potential of AMPK activation in the retina is strongly supported by the above studies, whereas our data show that the negative effect of AMPK activation in this form of RP is probably due to the inherent structural instability of P23H rhodopsin after rescue.

Consequently, this study highlights the need to focus our search for pharmacological chaperones for rhodopsin RP on allosteric correctors of misfolding, molecules unaffected by light that could stabilise the seven-transmembrane bundle of rhodopsin without affecting the function of cone opsins. Such molecules might need to target the intradiscal plug and not bind close to the retinal binding pocket, where most research efforts have centred. They also would need to achieve high concentrations in the retina (to match the level of mutant rhodopsin) with low toxicity and last for many days, as ROS rhodopsin takes 10 days to traffic to the RPE for degradation. Therefore, rather than improving mutant rhodopsin folding, therapeutic strategies might be better focused on clearing mutant rhodopsin and reducing its aggregation (22,23,64), as these homeostatic interventions could improve photoreceptor function and survival in animal models associated with toxic gain-of-function. Alternatively, RNA-based therapies could target mutant mRNA (65) or CRISPR/Cas9 gene editing could be used to target the mutation closer to its source.

Metformin is a relatively safe drug that is taken by over 20 million people worldwide. We have shown that it can stimulate the improved folding of a mutant protein that is normally misfolded and removed by ER-associated degradation. Therefore, metformin might be able to improve the folding of other mutant proteins that are normally degraded. This could be particularly useful in recessive conditions with amino acid substitutions that result in protein misfolding, degradation and loss of function (e.g. cystic fibrosis or recessive RP), where the restoration of some protein function might be sufficient to ameliorate disease. In dominant toxic gain-of-function diseases, however, reducing the ability of the protein quality control machinery to remove mutant proteins could exacerbate gain-of-function defects. As such, metformin could potentially exacerbate disease progression in rhodopsin RP patients with the P23H mutation and possibly other rhodopsin misfolding mutations, if they are prescribed for type II diabetes. This potential complication needs to be considered in terms of patient’s overall health and the risk to retinal function posed by uncontrolled diabetes, but other approaches might prove suitable for controlling patient’s diabetes without compromising retinal health.

## Methods

### Animals

All animal procedures were conducted according to the Home Office (UK) regulations, under the Animals (Scientific Procedures) Act of 1986, and with the approval of the local UCL Institute of Ophthalmology, London, UK ethics committee. The P23H-1 line rats were kindly provided by Professor Matt LaVail, (University of California San Francisco, USA, http://www.ucsfeye.net/mlavailRDratmodels.shtml) and crossed with wild-type Sprague Dawley (SD rats) to generate heterozygous P23H-1 rats. SD rats were purchased from Harlan (Blackthorn, UK). P23H KI mice were generated as previously described (14). Rhodopsin KO mice (*rho^−/−^*) were provided by Professor Peter Humphries (Trinity College, Dublin, Ireland). All animals were housed under a 12 h light (20–100 lux): 12 h dark (<10 lux) cycle, with food and water available *ad libitum*. Both sexes were used evenly for experiments.

### Drug treatments

P23H KI homozygous mice were moved to a dark cabinet at P0 and treated from P9 to P13 with either 300 mg/kg metformin (1,1-dimethylbiguanide hydrochloride, Sigma Aldrich, D5035) or vehicle. The intraperitoneal (IP) injections were carried out daily under red-light conditions and mice were humanely killed at P14 by cervical dislocation.

P23H-1 rats were treated from postnatal day P21 to P35 (5 weeks of age) or P21 to P49 (7 weeks of age); SD rats and rhodopsin KO mice were treated from postnatal day P21 to P35 P23H KI heterozygous mice from P21 to P45. Vehicle, 300 mg/kg (rats and *Rho^P23H/+ ^*mice) or 250 mg/kg (*Rho^-/-^* mice) of metformin was administered daily via IP injection. The dose was reduced for later mouse treatments because of potential general toxicity. Animals were monitored daily for any adverse effects and for body weight gain. At P36 or P49 rats were anaesthetised with ketamine/xylazine at 0.2 ml/100 g and mice with ketamine/domitor at 0.08 ml/10 g administered via an IP injection and were subjected to ERG and OCT before they were humanely killed by cervical dislocation.

### Electroretinography (ERG)

At P35 or P49, rats were dark-adapted overnight in a ventilated light-tight box. Scotopic electroretinogram tests were carried out under red-light conditions as described in (66). Briefly, pupils were dilated with topical 1% tropicamide (mice) or and 1% tropicamide and 2.5% phenylephrine hydrochloride (rats). Full-field scotopic ERG’s were obtained by using the Diagnosys system and Espion software (Diagnosys). Animals were dark-adapted for a further 10 min and simultaneous bilateral recording flash stimuli (10 ms to 1 ms duration, repetition rate 0.2 Hz) were presented via an LED stimulator (log intensity −5 to + 1) under scotopic conditions. For the Rho KO mice simultaneous bilateral recording flash stimuli (0.1 ms to 30 ms duration, repetition rate 0.2 Hz) were presented via an LED stimulator (log intensity -1 to + 3) under photopic conditions.

### Optical coherence tomography (OCT)

Animals were anaesthetised and their retinae were imaged using the Bioptigen Envisu R2300 Spectral-domain ophthalmic imaging system (SDOIS). To prevent cornea desiccation during the procedure, topical Systane Ultra (Alcon) lubricant eye drops were applied bilaterally every minute. Following lubrication, the rats were positioned for imaging in the animal imaging mount-rodent alignment stage (AIM-RAS). The front teeth of the rat were gently placed over the bite bar and a band was placed over the nose to ensure restriction of movement. The animals were positioned so that the images were centred to the optic nerve head. Images were acquired by the InVivoVue Clinic application using a rectangular scanning protocol consisting of a either a 2 mm by 2 mm perimeter with 750 A-scans (lines) and 5 B-scans (frames) with a 60 frames/B-scan (for rat retina) or 1.4 mm by 1.4 mm perimeter with 750 A-scans (lines) and 10 B-scans (frames) with a 20 frames/B-scan (for mouse retina). All OCT images were averaged using the InVivoVue software to obtain a better image resolution. Following OCT analysis rats were humanely killed by cervical dislocation. The Bioptigen InVivoVue Diver 2.0 was used to enable manual segmentation of the retinal layers and the outer nuclear layer thickness was measured after exporting results from Diver to Excel.

### Western blotting and sedimentation assay

Retinae were extracted and lysed with ice-cold RIPA buffer (50 mM Tris-HCl pH8, 150 mM NaCl, 1 mM EDTA, 1% NP-40, 0.1% SDS and 0.05% sodium deoxycholate) containing 2% (v/v) mammalian protease inhibitor cocktail 2% (v/v) mammalian phosphatase inhibitor cocktail (Sigma Aldrich). Retina lysates were sonicated for 2 × 30 s centrifuged for 15 min at 12,000 × g and at 4ºC and diluted in sodium dodecyl sulfate (SDS) sample loading solution (0.0625 M Tris pH 6.8, 2% (w/v) SDS, 30% (v/v) glycerol, 20% (v/v) β-mercaptoethanol). The rhodopsin sedimentation assay in P23H-1 rat retinae was performed as previously described (22). For cellular studies, cell lysates were prepared as previously described (67). Samples for rod opsin were not heat denatured, whereas samples for all the other antigens were heated at 95ºC for 5 min before resolving by SDS-polyacrylamide gel electrophoresis (SDS-PAGE) and western blotting. Proteins were transferred to nitrocellulose in 25 mM Tris, pH 8.3, 192 mM glycine, 0.01% SDS (w/v) and 20% (v/v) methanol. To prevent non-specific binding, membranes were first blocked at 4ºC with either 5% (w/v) Marvel Milk in PBS with 0.1% (v/v) Tween buffer, pH 7.5 or 5% (w/v) bovine serum albumin (BSA) in TBS, pH 7.5, with 0.1% (v/v) Tween buffer. Immunodetection of proteins of interest was carried out with the following primary antibodies: mouse anti-Rho-1D4 (1:1000 from Professor Robert Molday (Department of Biochemistry and Molecular Biology, University of British Columbia, Canada), mouse anti-β-tubulin (1:3000;clone TUB 2.1, T4026, Sigma Aldrich), rabbit anti-phospho-AMPKα (Thr172) (1:1000, 40H9, 2535S, New England Biolabs (NEB), anti-rabbit AMPKα (1:1000, D63G4, 5832S, NEB), anti-phospho-S6 Ribosomal Protein (Ser235/236), (p-rpS6 Ser ^235/236^), (1:1000, 4858, NEB), anti-phospho-S6 Ribosomal Protein (Ser240/244) (p-rpS6 Ser ^240/244^), (1:1000, 5364, NEB), anti-phospho-p70 S6 Kinase (Thr389), (p-p70S6K ^Thr 389^) (1:1000, 108D2, 9234, NEB), p70 S6 Kinase (1:1000, 49D7, 2708, NEB), 4E-BP1 (1:1000, 53H11, 9644, NEB), 4EBP1 (1:1000, 53H11, 9644, NEB), anti-actin (1:2000, Millipore), PERK (1:1000, NEB), eIF2a (1:1000, NEB), BiP (1:2000, Sigma Aldrich), anti-phospho eIF2α (pSer51) (1:1000, Sigma Aldrich), IRE1 and anti-phospho IRE1 (1:1000, NEB), ATF6 (1:1000, Santa Cruz). Blots were probed with either goat anti-rabbit (1:30000; 32460, ThermoFisher Scientific) or anti-mouse (1:50000; 32430, ThermoFisher Scientific) secondary antibodies. Proteins were detected with the ECL Plus reagent (GE Healthcare) or ECL prime (VWR).

### Immunohistochemistry

For outer segment measurement studies, tissue was prepared as previously described (23). Semi-thin and ultra-thin sections were cut with a Leica Ultracut S microtome fitted with an appropriate type of diamond knife. For light microscopy semi-thin sections were stained with 1% toluidine blue and 1% borax in 50% ethanol. Ultra-thin sections were treated for 5 min with 1% uranyl acetate in 50% ethanol followed by Reynold’s lead citrate, prior to viewing and photography with a JEOL 1010 TEM operating at 80 kV.

For fluorescence studies, eyes were collected at specified time points and fixed overnight in 4% paraformaldehyde at 4 °C. Post-fixation eyes were cryoprotected by incubation in 30% sucrose in PBS. Eyes were then frozen and cyrosectioned as previously described (22). Cryosectioned eyes were stained with mouse anti-Rho-1D4 (1:1500), rabbit anti-calnexin (1:600; C4731, Sigma Aldrich) and rat anti-prominin-1 (1:200; clone 13A4, CD133, eBioscience) primary antibodies in blocking solution (3% BSA, 10% normal goat serum) and visualised with Alexa Fluor 488 (1:1000; A11001/A11005, Life Technologies Ltd) and 594 (1:1000; A11007, Life Technologies Ltd) conjugated IgGs. 4’,6-diamidino-2-phenylindole dihydrochloride (DAPI) (Sigma Aldrich) staining was used to visualise the nuclei.

### Fluorescence imaging and analysis

Retina images were obtained using a Carl Zeiss LSM700 laser-scanning confocal microscope whereas images for nuclei/column and ROS length analysis were taken on a Carl Zeiss LSM510 confocal microscope. Images were exported with Zen 2009 software were then prepared using Adobe Photoshop and Illustrator CS4. All measurements were performed in ImageJ (http://rsbweb.nih.gov/ij/). The Pearson's and Mander's co-localisation co-efficients in cells co-stained using Rho-1D4 and either anti-calnexin or anti-prominin-1 was measured with JaCOP plug-in and ImageJ software. The relative amounts of rhodopsin present in treated and untreated OS were assessed by comparing the fluorescent intensity measured in line scans generated using fixed detector gain and offset settings previously set up to yield non saturated images of the brightest treated specimens. The hardware was set up to acquire 2K images at 1 Airy unit resolution (0.8um @ l488 nm, NA= 1.4). An intensity distribution across the Z axis of the line scan was then generated using the ImageJ profile tool and the maximum intensity across the ROS averaged.

### Cell culture, transient transfection and drug treatments

SK-N-SH human neuroblastoma cells were maintained and transfected essentially as previously described (68). The WT-GFP and P23H-GFP rod opsin plasmids in pEGFP-N1 vector were prepared as previously described (6). After 3 h transfection and 2 h recovery in serum, cells were either left untreated or treated with 1 mM metformin or 2 mM AICAR (Sigma Aldrich, A9978) for 18 h at 37ºC. HEK293S human embryonic kidney cells provided by J. Nathans (John Hopkins School of Medicine) were maintained and transfected essentially as previously described (46). HEK293S cells expressing the WT or P23H mutant bovine rod opsin genes were treated with 11-*cis* retinal provided by Rosalie K Crouch (Storm Eye Institute, Medical University of South Carolina) during receptor biosynthesis as described in (46) except that 10 µM of 11-*cis* retinal (final concentration) was added as one installment in the dark. Metformin also was added only once to cells at a final concentration of 300 µM. Cells were harvested 24 h post treatment.

### HEK 293S stable cell lines

Tetracycline-inducible WT and P23H mutant rod opsin genes were constructed by previously described procedures (38). Opsin expression was induced by the addition of 2 μg/ml tetracycline after which 11-*cis* retinal (10 µM), metformin (300 µM) or AICAR (2 mM) treatments were immediately initiated.

### Rod opsin cell surface expression by in-cell western

SK-N-SH cells were seeded in 24-well plates and transfected with HA-WT or HA-P23H rod opsin a gift of Professor Yuichi Hashimoto (The University of Tokyo) (69). Three hours after transfection and 2 h after recovery in serum, cells were either left untreated or treated with 1 mM metformin, 2 mM AICAR, 100 nM and 300 nM cycloheximide (CHX, Sigma Aldrich, C7698) or 100 and 300 nM emetine (Sigma Aldrich, E2375). Following 18 h treatment at 37ºC, cells were washed with PBS and fixed in 4% PFA for 10 min. Cells were permeabilised with 0.25% Triton X-100 for 10 min to assay total cellular rod opsin levels or left unpermeabilised to detect rod opsin cell surface expression. Cells were then blocked with 3% BSA and 10% normal goat serum for 1.5 h, followed by incubation with anti-HA antibody (1:500, H6908, Sigma Aldrich) for 1 h. Cells were then washed with PBS and incubated with IRDye 680 secondary antibody (1:1000, LI-COR) for 1 h protected from light. Cells were washed and fluorescent signal measured using a LI-COR Odyssey plate reader.

### Lactate dehydrogenase (LDH) cell death assay

The LDH assay was performed in SK-N-SH cells essentially as previously described (70) starting 48 h after the addition of 1 mM metformin or 2 mM AICAR following the manufacturer’s instructions.

### Rod opsin internalisation assay

SK-N-SH cells were seeded in 24-well plates and transfected with HA-WT or HA-P23H rod opsin. Three hours after transfection and 2 h recovery in serum, cells were either left untreated or treated with 1 mM metformin. Following 18 h treatment live cells were incubated with a mouse HA antibody (1:500, H6908, Sigma Aldrich) or HA and metformin in culture media at 37ºC to label rod opsin at the cell surface. Following 30 min incubation cells were washed with PBS, the medium was replaced to allow rod opsin endocytosis and the cells incubated for 0, 1 and 3 h. Cells were fixed in 4% PFA, were not permeabilised in order to detect exclusively rod opsin cell surface expression and blocked with 3% BSA and 10% normal goat serum for 1 h followed by incubation with IRDye 680 secondary antibody (1:1000, LI-COR) for 1 h protected from light. Cells were then washed and fluorescent signal measured using a LI-COR Odyssey plate reader.

### Immunofluorescence in cells

Immunofluorescence assays were carried out as previously described (70). The mouse anti-Rho-4D2 (1:300; MABN15) antibody was from Millipore. Fluorescence was observed with a Carl Zeiss LSM 700 confocal laser-scanning microscope for image acquisition. Images were exported from the LSM browser to Adobe Photoshop for figure preparation and annotation by Adobe Illustrator.

### Immunoaffinity purification of rhodopsin

The procedure for purification of rhodopsin pigments from detergent solubilised HEK293S cell extracts involving Rho-1D4 Sepharose 4B (Amersham Biosciences) has been described in (46). Rhodopsin was eluted in 10 mM BTP (Sigma Aldrich) at pH 7.2 containing 100 µM 9 mer peptide (TETSQVAPA) corresponding to the rhodopsin C-terminus (Peptide Protein Research).

### UV-visible absorption spectroscopy and photobleaching assay of rhodopsin

Procedures for UV-visible absorption spectroscopy of rhodopsin and its photobleaching have been described in detail (46).

### Thermal stability of rhodopsin and MII stability of rhodopsin

Assays of the thermal stability of rhodopsin at 37 °C were carried out essentially as described in (46) except that the UV-visible absorption spectra were acquired at 30-min intervals over 4 h. Similarly, measurements of the rate of MII decay in rhodopsin also were described in (46).

### Statistical analyses

The values for all the experiments represent the means ± SEM. The exact number of samples is described in greater detail in the figure legends, as it was dependent on experimental design. All statistical analyses were performed with the unpaired two-sided Student's *t* test. A *P* value of less than 0.05 was considered statistical significant.

## Supplementary Material

Supplementary Material is available at *HMG* online.

## Supplementary Material

Supplementary Data
