# Supplementary material for: Rescue of mutant rhodopsin traffic by metformin-induced AMPK activation accelerates photoreceptor degeneration
Source: Hum Mol Genet. 2016 Nov 30;26(2):305–19. doi: 10.1093/hmg/ddw387 (PMC5351934; doi:10.1093/hmg/ddw387)
Supplement: Supplementary Data [file ddw387_Supp.pdf]

Supplementary Material

Rescue of mutant rhodopsin traffic by metformin-induced AMPK activation accelerates photoreceptor degeneration Athanasiou et al

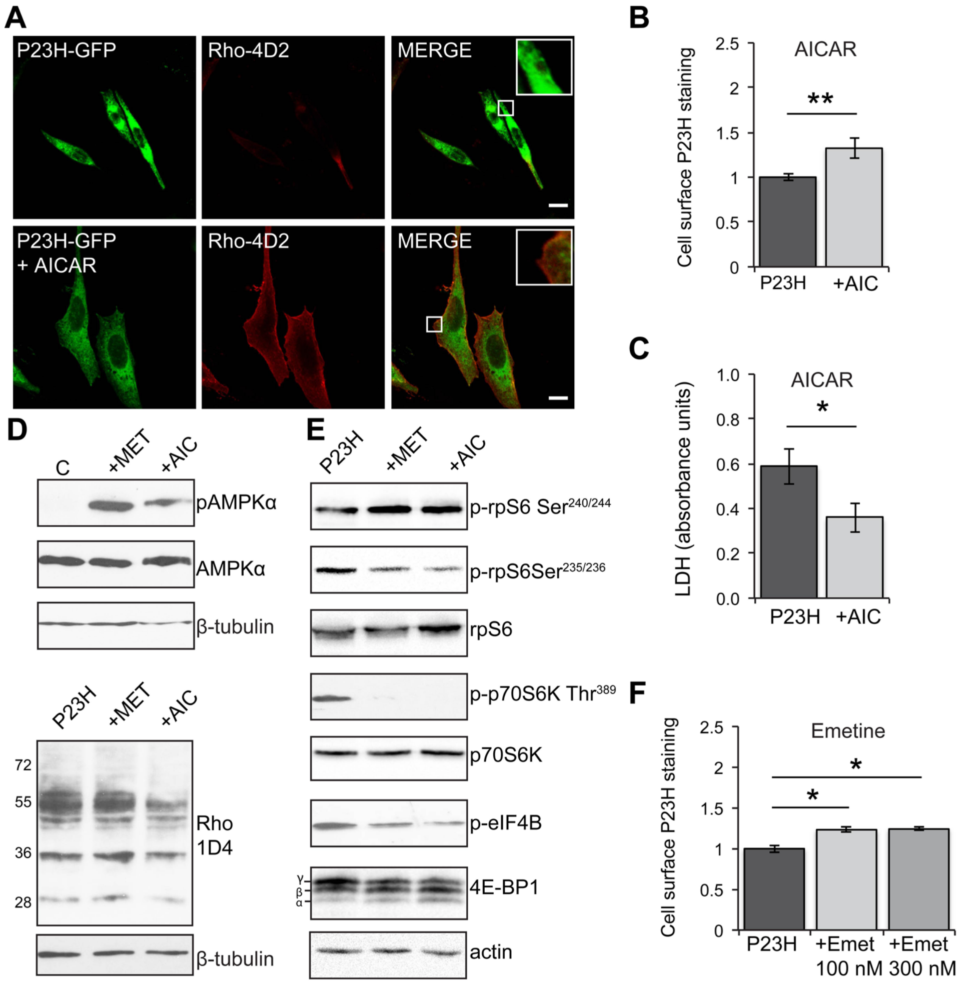

Supplementary Figure 1. ***AICAR improves P23H rod opsin folding and reduces P23H related cell death***. (**A**) SK-N-SH cells were transfected with P23H-GFP rod opsin (green) and treated with AICAR (2 mM), as indicated. After 18 hours of treatment, fixed, non-permeabilised cells were stained with Rho-4D2 antibody against the extracellular N-terminus of rod opsin (red). Cells were analysed by confocal microscopy under identical scanning conditions. Scale bar: 10 μm. Boxed regions on the right show rod opsin localisation at the plasma membrane at high magnification. (**B**) In-cell western analysis on HA-P23H rod opsin SK-N-SH cells untreated or treated with 2 mM AICAR (+AIC) for 18 hours. Cells were fixed and immunostained with an HA antibody against the extracellular N-terminus of rod opsin. The non-permeabilised (cell surface) immunoreactivity was determined as a percentage of total permeabilised immunoreactivity. The data were normalised to untreated HA-P23H, values ± SEM, n≥4, **p<0.01, unpaired two-sided Student's *t* test. (**C**) LDH assay for P23H-GFP in the presence of AICAR (+AIC), as indicated, values ± SEM, n≥3, *p<0.05, unpaired two-sided Student's *t* test. (**D**) Extracts from control (C) SK-N-SH cells and cells treated with 1 mM metformin (+MET) or 2 mM AICAR (+AIC) were western blotted with an anti-phospho-AMPKα (p-AMPKα) or an anti-AMPKα antibody, as indicated. Untreated- and metformin- or AICAR-treated P23H-GFP cells were blotted with the Rho-1D4 antibody. β-Tubulin was used as a loading control. (**e**) Cell lysates were western blotted with antibodies against rpS6, p70SK6, eIF4B and 4E-BP1 as indicated, actin was used as a loading control. (**F**) In-cell western analysis on HA-P23H rod opsin SK-N-SH cells untreated or treated with emetine 18 hours. The data were normalised to untreated HA-P23H, values ± SEM, n≥4, *p<0.05, unpaired two-sided Student's *t* test.

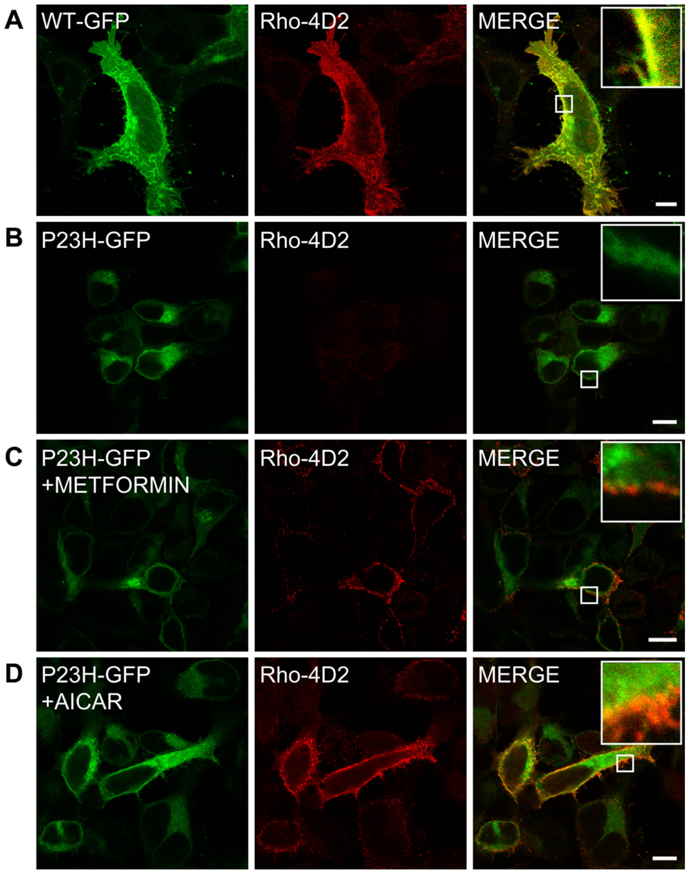

Supplementary Figure 2. ***Metformin and AICAR improve P23H rod opsin traffic in HEK293S cells*** HEK293S tetR cells expressing (**A**) WT-GFP rod opsin (green) and (**B**) P23H-GFP rod opsin (green) in the absence of any treatment or treated with (**C**) metformin (300 μM) or (**D**) AICAR (2 mM), as indicated. After 18 hours of treatment, fixed, non-permeabilised cells were stained with Rho-4D2 antibody directed against the extracellular N-terminus of rod opsin (red). Cells were analysed by confocal microscopy at the same settings. Scale bar: 10 μm. Boxed regions on the right show rod opsin localisation on the plasma membrane at high magnification.

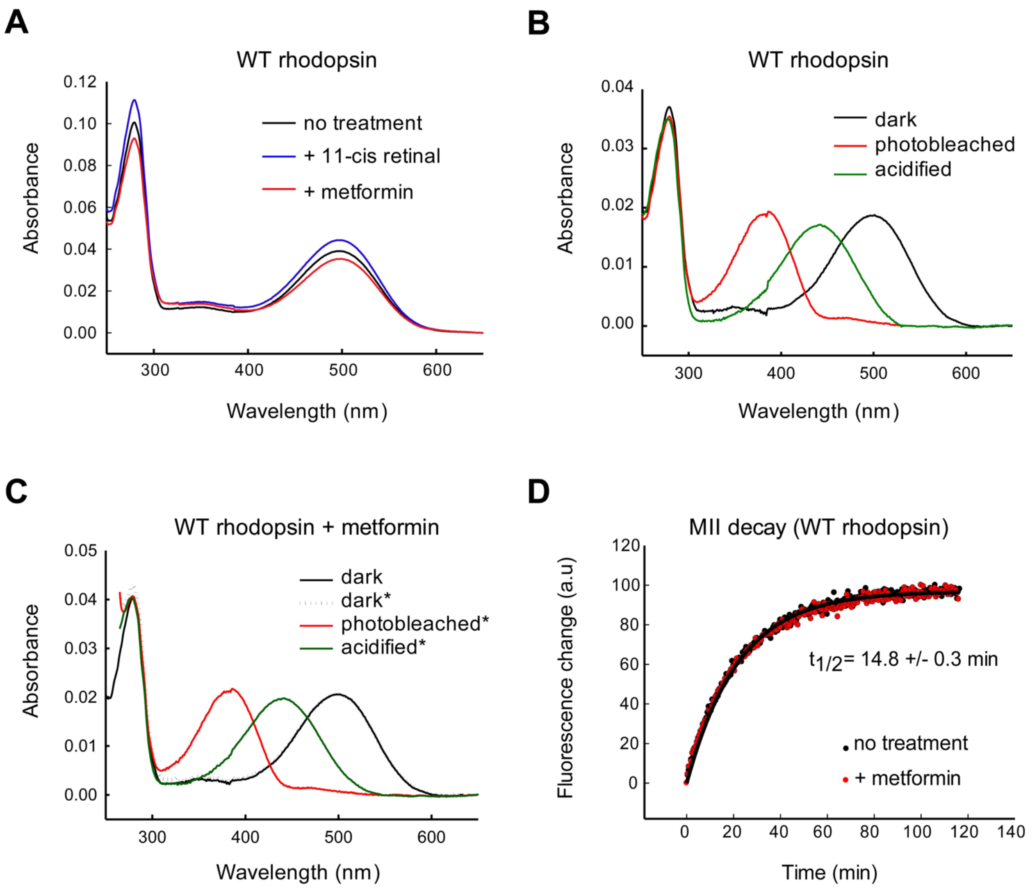

Supplementary Figure 3. ***Metformin does not affect spectral properties WT rhodopsin pigment*** (**A**) UV-visible absorbance spectroscopy of purified WT pigment from transiently transfected HEK-293S cells in the absence (black trace) or presence of 11-*cis* retinal (blue trace) or metformin (red trace) during opsin biosynthesis. (**B-C**) Photobleaching profile of WT rhodopsin pigment in the absence (**B**) or presence (**C**) of metformin (300 μM) added directly to the purified pigment. In both cases, UV-visible absorbance spectroscopy of pigment was first scanned in the dark (dark or dark*) before illuminating pigment sample with a 150W fiber optic light that is fitted with a >495 nm filter for 30 s and taking another spectrum immediately after photobleaching (red). *Indicates pigment obtained from cells treated with metformin during biogenesis. Upon completion of photobleaching, 4N H_2SO_4 was added to each sample to create a protonated shift base (green), confirmed by a 440 nm absorbance trace. (**D**) MII stability of WT rhodopsin in the absence (black) or presence of metformin (red). Purified pigment was illuminated as described above and the relative increase in tryptophan fluorescence due to loss of 11-*cis*-retinal (a fluorescence quencher) from the binding pocket was measured by fluorescence spectroscopy.

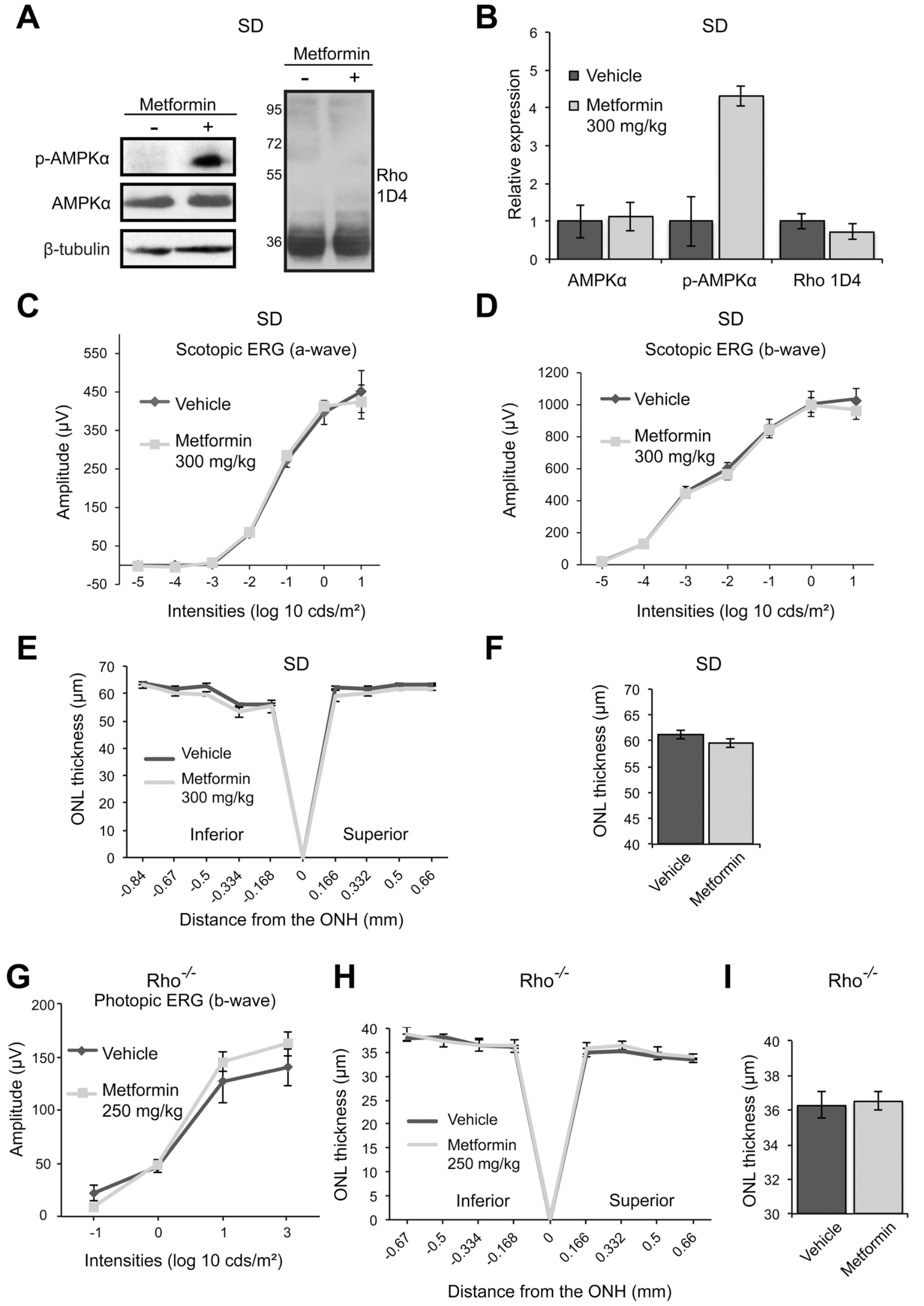

Supplementary Figure 4. ***Metformin does not affect photoreceptor function and survival in control SD rats and rhodopsin KO mice.*** SD rats were treated from P21-P35 with either 300 mg/kg metformin or vehicle-PBS. Metformin and vehicle were administered daily via IP injection. (**A**) Retinae of metformin (+) or vehicle-treated (-) SD rats were western blotted with p-AMPKα or an anti-AMPKα antibody to confirm the activation of AMPKα protein in the retina after metformin treatment and anti-Rho 1D4 antibody, as indicated. β-Tubulin was used as a loading control. (**B**) Densitometric analysis was used to calculate the levels of p-AMPKα, AMPKα and rhodopsin relative to vehicle after normalisation to β-tubulin; values are means ± SEM, n≥3. **(C-D**) Scotopic ERG responses, a-wave (**C**), b-wave (**D**) of SD rats (P36) treated with either 300 mg/kg metformin (n=5 biological replicates) or vehicle-PBS (n=6 biological replicates). Values are means ± SEM. (**E, F**) Wild-type SD ONL thickness at P36 after two weeks of treatment with 300 mg/kg metformin (n=5 biological replicates) or vehicle-PBS (n=5 biological replicates) as assessed by OCT measurements. Results were either expressed as a spider plot from optic nerve head (ONH) (**E**) or as mean ONL thickness across the whole retina (**F**). Values are means ± SEM. (**G**) Photopic ERG responses (b-wave) of rhodopsin KO (*Rho*$^{-/-}$) mice (P36) treated with either 250 mg/kg metformin

(n=5 biological replicates) or vehicle-PBS (n=4 biological replicates). Values are means ± SEM. (**h-i**) Rhodopsin KO ONL thickness at P36 after two weeks of treatment with 250 mg/kg metformin (n=5 biological replicates) or vehicle-PBS (n=4 biological replicates) as assessed by OCT measurements. Results were either expressed as a spider plot from optic nerve head (ONH) (**H**) or as mean ONL thickness across the whole retina (**I**).
